# Intravenous anti-*P. aeruginosa* IgY antibodies increase bacterial concentration in porcine septic shock with bacteremia

**DOI:** 10.1186/s40635-026-00952-y

**Published:** 2026-08-19

**Authors:** Alexander Otterbeck, Paul Skorup, Anders Larsson, Johan Stålberg, Maria Baumgarten, Maria Lampinen, Hans Hjelmqvist, Miklós Lipcsey

**Affiliations:** 1 Anesthesiology and Intensive Care, Department of Surgical Sciences, Uppsala University, entrance 70, 1 floor, Uppsala, SE-751 85 Sweden; 2 Section of Infectious Diseases, Department of Medical Sciences, Uppsala University, Uppsala, Sweden; 3 Section of Clinical Chemistry, Department of Medical Sciences, Uppsala University, Uppsala, Sweden; 4 Section of Infection Medicine, Department of Clinical Sciences, Lund University, Lund, Sweden; 5 Hedenstierna Laboratory, Department of Surgical Sciences, Uppsala University, Uppsala, Sweden; 6 Department of Anaesthesia and Intensive Care, Faculty of Medicine and Health, Örebro University, Örebro, Sweden

**Keywords:** Septic shock, Immunoglobulin Y, *Pseudomonas aeruginosa*

## Abstract

**Background:**

Bacteremia caused by *Pseudomonas aeruginosa* carries a high mortality rate. *P. aeruginosa* also possesses several resistance mechanisms to antibiotics, mandating studies of novel therapies. Specific polyclonal anti-*P. aeruginosa* IgY-antibodies (*Pa*-IgY) have been reported to decrease *P. aeruginosa* concentration in the airways. In this study, we investigated the effects of intravenous *Pa*-IgY on *P. aeruginosa* septic shock.

**Method:**

Pigs were anaesthetized, mechanically ventilated, and allocated to receive either intravenous *P. aeruginosa* only, *P. aeruginosa +* low dose *Pa-*IgY, *P. aeruginosa +* high dose *Pa-*IgY or high dose *Pa-*IgY only. The experiment lasted 6 h with repeated blood cultures, blood tests, monitoring of physiological parameters and postmortem organ cultures. We performed in vitro cultures of *P. aeruginosa* +/- porcine neutrophils and +/- *Pa-*IgY and flow cytometry of FITC-marked inactivated *P. aeruginosa* cultured with porcine blood +/- *Pa-*IgY.

**Results:**

The pigs developed septic shock with accompanying respiratory failure as a result of the *P. aeruginosa* infusion. Treatment with *Pa-*IgY increased bacterial concentrations, both in the porcine model in blood (*p* = 0.004) and when *P. aeruginosa* was cultured after pre-treatment with *Pa-*IgY +/- neutrophils in vitro (*p* < 0.001). The phagocytosis of *P. aeruginosa* in porcine blood was similar with and without *Pa-*IgY. *Pa-*IgY had no effect on the development or severity of septic shock. Animals with high dose *Pa-*IgY had higher PaO_2_/FiO_2_-ratio and static compliance. The inflammatory response was similar apart from higher IL-10 in animals treated with *Pa-*IgY (*p* = 0.007).

**Conclusion:**

In pigs with *P. aeruginosa* septic shock, treatment with *Pa-*IgY was associated with higher bacterial concentration in blood without an increased inflammatory response.

**Supplementary Information:**

The online version contains supplementary material available at 10.1186/s40635-026-00952-y.

## Background

*Pseudomonas aeruginosa* is a pathogen known to cause difficult to treat infections in severely ill patients. Bloodstream infections caused by *P. aeruginosa* in intensive care unit (ICU) patients have a high mortality rate of approximately 50% [1]. Further, resistance to several broad-spectrum antibiotics is common [2]. When caused by multidrug resistant pathogens, mortality from *P. aeruginosa* infections increases dramatically [3, 4]. Therefore, *P. aeruginosa* is listed as a priority pathogen for Research and Development of new antibiotics by the World Health Organization [5].

Immunoglobulin Y (IgY) is the primary serum antibody in birds and reptiles [6]. The inexpensive production of specific polyclonal anti-P. *aeruginosa* antibodies (*Pa-*IgY) consists of inoculating hens with *P. aeruginosa* and then harvesting antibodies from the egg yolks [7]. The anti-bacterial mechanism of *Pa*-IgY has primarily been studied in vitro. *Pa-*IgY opsonize pathogens by binding to surface antigens (such as flagellum on *P. aeruginosa*) which increases bacterial clearance by neutrophils [8, 9]. The inflammatory pathways activated by *P. aeruginosa* increase levels of of tumor necrosis factor-α (TNF-α), interleukin-6 (IL-6), interleukin-10 (IL-10), and interleukin-8 (IL-8) [10, 11]. Data from both clinical- and experimental *P. aeruginosa* studies show an antibacterial effect of *Pa-*IgY [6, 12, 13]. The study of *Pa-*IgY as a potential treatment for bacteremia caused by *P. aeruginosa* has not been studied in vivo. This also provides a possibility to study the effect of *Pa-*IgY on the inflammatory response in a large mammal with immunological similarities to humans.

We hypothesized that intravenous treatment with *Pa-*IgY lowers blood concentrations of *P. aeruginosa* in piglets. Pigs are physiologically, immunologically and anatomically similar to humans and therefore represent a research animal well suited for this experimental study. The use of advanced physiological monitoring and drawing of repeated blood samples is possible thank to the size and anatomy of pigs [14, 15]. This model also allows for invasive monitoring and repeated blood sampling.

Our primary endpoint was group difference in concentration of *P. aeruginosa* in blood over time in pigs treated with *Pa-*IgY vs. *placebo*. Secondary endpoints were group differences in organ function, inflammatory markers, neutrophil activity, organ inflammation and organ growth of *P. aeruginosa*. We also conducted ex vivo experiments to study the ability of *Pa-*IgY to stimulate phagocytosis by porcine neutrophils in whole blood.

## Materials and methods

### Ethical approval

Animals were handled according to guidelines from the Animal Ethics Board (Uppsala, Sweden) and the European Union’s directives for animal research. Ethical approval was granted by the Uppsala animal ethics committee (application 5.8.18–08592/2019). When applicable, MQTiPSS-recommendations have been adhered to [16]. Data is presented according to ARRIVE-guidelines when relevant [17].

### Study protocol

This study is a *placebo* controlled experimental animal study. A cross-breed between Swedish Landrace, Yorkshire, and Hampshire pigs, 6–8 weeks old, from a breeder outside of Uppsala, was used. The experiments were carried out in an ICU-setting at the Hedenstierna laboratory (Uppsala, Sweden), staffed by experienced lab technicians. Pigs arrived in the morning. After approximately 2 h of preparation including a stabilization period, experiments were started and lasted for 6 h. Pigs were excluded before randomization if there were clinical signs of infection, respiratory or hemodynamic illness.

Anaesthesia and supportive care were provided according to previously published methods [18]. Breifly, animals were anaesthetized, received a tracheostomy and mechanical ventilation. Vascular- and urinary catheters were placed, including a pulmonary artery catheter. 750 mg of perioperative Cefuroxime (to which *P. aeruginosa* is naturally resistant) was administered to all pigs. Clinical signs of inadequate anaesthesia or a withdrawal reflex when exposing the soles to a painful stimulus prompted additional analgesics and sedatives.

Noradrenaline 20 µg/mL (Noradrenalin, Hospira Nordic, Stockholm, Sweden) was started as a continuous infusion at 5 mL/h if needed and increased accordingly to maintain a mean arterial pressure (MAP) > 60 mmHg. At cardiac output < 2 L/min, a clinical decision was made to either increase the rate of noradrenaline or to give a 15 mL/kg bolus of Ringer’s Acetate. In the case of life-threatening hemodynamic collapse, 1 mL boluses of adrenaline 10 µg/mL (Adrenalin, Martindale Pharma. Ethypharm, Saint-Cloud Cedex, France) were administered intravenously. At the end of the experiment, the pigs were sacrificed by injection of 20 mL KCl.

### IgY-production

*Pa-*IgY was produced according to previously published protocols [6, 19, 20]. Briefly, hens were inoculated with *P. aeruginosa* and *Pa-*IgY was then purified from the egg yolks. Equal anti-*P.aeruginosa* activity between batches was ensured by ELISA. The resulting concentration was approximately 22.5 mg/mL. *Pa*-IgY was donated by IgY Lab Systems AB (Uppsala, Sweden).

### *P. aeruginosa*

The strain of *P. aeruginosa* (PA-103, ATC 29260, CCUG31589) used is a clinical isolate from sputum with a type 3 secretion system and exotoxin A production. The logarithmic growth phase of the strain in vitro was determined to be 105 min and the strain is resistant to pig serum. *P. aeruginosa* was cultured overnight on cysteine-lactose-electrolyte-deficient agar (CLED) plates at 37 °C, and in the morning, the colonies were transferred to Lysogeny broth and cultured until log-phase. The solution was then centrifuged (20 min at 4000 RPM) and the bacterial pellet dissolved and diluted in NaCl 9 mg/mL until 1.0 OD_600 nm_. This corresponded to an average concentration of 0.93 ± 0.22 × 10^9^ CFU/mL when cultured overnight at 37 °C on CLED plates.

### Intervention

Pigs were allocated to receive either intravenous *P. aeruginosa* (*Pa*, *n* = 7) + 100 mL NaCl 9 mg/mL (*placebo)*, *P. aeruginosa* + 1 mL *Pa-*IgY 22.5 mg/mL (*Pa* + 1 *Pa-*IgY, *n* = 7), *P. aeruginosa* + 100 mL *Pa-*IgY 22.5 mg/mL (*Pa* + 100 *Pa-*IgY, *n* = 7), 100 mL *Pa-*IgY (100 *Pa-*IgY only, *n* = 3) or anaesthesia only (*n* = 7) (see Table 1). *P. aeruginosa* was given as an initial bolus of 10 mL followed by an infusion at a rate of 0.3 mL/kg/h for 1 h. *Pa-*IgY or *placebo* was given as a bolus immediately after *P. aeruginosa*.

**Table 1:** Overview of experimental groups, treatment per group and number of pigs per group

Group	Treatment	Number of pigs
*Pa*	*P. aeruginosa* i.v.	7
*Pa* + 1 *Pa-*IgY	*P. aeruginosa* + 1 mL *Pa-*IgY 22.5 mg/mL i.v.	7
*Pa +* 100 *Pa-*IgY	*P. aeruginosa* + 100 mL *Pa-*IgY 22.5 mg/mL i.v.	7
100 *Pa-*IgY only	100 mL *Pa-*IgY 22.5 mg/mL i.v.	3
Anaesthesia only	No treatment	7

### Data collection

We measured physiological parameters, collected arterial and mixed venous blood gases, collected blood for blood cultures, and collected blood samples in EDTA-, sodium citrate- and lithium heparin tubes at baseline and every hour during the experiment. We collected urine at the end of the experiment and organ biopsies after termination.

Heart rate (HR) was recorded from a 3-lead ECG, mean arterial pressure (MAP) from a carotid arterial line, cardiac index (CI, via thermodilution), mean pulmonary arterial pressure (MPAP), and core temperature from a pulmonary artery catheter. Central venous pressure was measured through a central venous catheter to calculate systemic vascular resistance index (SVRI)[SVRI = 80 x (MAP-CVP)/CI]. Global delivery of oxygen (DO_2_) was calculated by: DO_2_ = cardiac output x [hemoglobin] x 1,34 x SaO_2_ + 0,023 x PaO_2_. Static compliance was measured after a three second inspiratory hold.

Blood gases were analyzed in blood gas analyzers at the animal research facility (ABL835- FLEX Radiometer and OSM3 Oximeter Radiometer). Blood samples were analyzed for complete blood counts, plasma creatinine levels, bilirubin levels and plasma cytokines including IL-6 (detection limit > 120 pg/mL), TNF-α (detection limit > 25 pg/mL) IL-10 (detection limit > 20 pg/mL), IL-8 (detection limit > 120 pg/mL) and IL-1β (detection limit > 60 pg/mL). Cytokines were analyzed using commercial ELISAs for porcine IL-6, TNF-α, IL-10 and IL-8 (DY686, DY690B, DY693B, DY535 and DY681 R&D Systems, Minneapolis, MN, USA). Plasma was analyzed for anti-*P. aeruginosa* activity using ELISA, which is a measure of the degree of binding by antibody to *P. aeruginosa* (cut-off for background activity < 0.5 arbitrary units) [21]. The analysis uses anti-chicken antibodies to detect anti-*P. aeruginosa* binding and therefore only reacts to *Pa-*IgY. The analysis of anti-*P. aeruginosa* activity was not performed in the anaesthesia only group. Blood was drawn from the arterial catheter and 100 µL was cultured on CLED plates in duplicate. Post mortem, a thoracotomy and laparotomy were performed and sterile biopsies of the right lower pulmonary lobe, the distal spleen and the left lateral liver lobe were collected. The biopsies were weighed and then crushed in a pestle and mortar. The biopsies were then dissolved in 3 mL NaCl 9 mg/mL and 200 µL of the solution was cultured on CLED plates in duplicate, undiluted and diluted both 10 and 100 times. All cultures were incubated overnight at 37 °C. The bacterial concentration of all cultures in this study was determined with the viable count technique [22]. Briefly, the lowest dilution with a colony count of 30–300 colonies was used to determine the bacterial concentration of the sample.

### Ex Vivo phagocytosis

The strain used in the in vivo experiments was inactivated and labeled with fluorescein isothiocyanate (FITC) using a methodology previously described [23]. A detailed description of the methodology and results can be found in the supplement. Briefly, FITC-*P. aeruginosa* was incubated for one hour with different dilutions of *Pa-*IgY for opsonization. Porcine blood was added and reincubated for one hour for phagocytosis to occur. The degree of phagocytosis was then estimated by measuring the fluorescence of granulocytes using flow cytometry.

### Culture of neutrophils, *P. aeruginosa* and *Pa*-IgY

Porcine neutrophils were isolated from blood using Polymorphprep™ (Axis-Shield PoC AS, Oslo, Norway). One hundred µL of bacterial solution containing *P. aeruginosa* 10^5^ CFU/mL (the concentration was determined in pilot experiments where an effect of *Pa-*IgY was seen) and 100 µL of solution containing *Pa-*IgY were incubated together at 37 °C for 1 h under agitation for opsonization to occur. To test different *P.* aeruginosa:*Pa-*IgY ratios, dilutions of *Pa-*IgY in steps of 10 was used, from 10 to 10 000 000 times. One hundred µL of this solution was incubated again at 37 °C with 40 µL of the neutrophil solution for 2 h for neutrophil activation and phagocytosis to occur.

The samples were cultured on CLED-plates at 37 °C overnight. We cultured samples containing *P. aeruginosa* only, *P. aeruginosa* + neutrophils, *P. aeruginosa* + neutrophils + *Pa-*IgY and *P. aeruginosa* + *Pa-*IgY. NaCl was added as a control substance to avoid dilution effects. The experiment was performed in triplicate, each experiment using neutrophils from a new pig. We also performed electron microscopy of the solution of neutrophils, *P. aeruginosa* and undiluted *Pa-*IgY described above. Methodology and results of electron microscopy can be found in the supplement.

### Histopathological analysis

Post-mortem biopsies of the right lower pulmonary lobe, distal spleen, and the left lateral liver lobe of 5 pigs in the *Pa*-group, 5 pigs the *Pa* + 100 *Pa-*IgY group were secured. Biopsies from 1 pig in the anaesthesia only group, 1 pig in the *Pa +* 1 Pa-IgY group and 1 pig from the *Pa-*100 IgY group were also saved. Further details of the method for histopathological analysis can be found in the supplement.

### Statistical analysis

This exploratory study, using a severe large animal model in a controlled experimental setting, was designed to detect a large effect in the primary outcome. In accordance with the principles of 3R, seven animals per main experimental group (*Pa*,* Pa* + 1 *Pa*-IgY and *Pa* + 100 *Pa-*IgY) were considered sufficient. The use of repeated measures and supplemental in vitro experiments also allowed reduction in the number of animals. Three pigs were included in the 100 *Pa*-IgY only group as this group served to identify major adverse effects for future study and anti-*P. aeruginosa* activity assay interpretation, not for formal analysis of secondary outcomes.

Data is presented according to normality (assessed in a histogram and tested using Shapiro-wilks test, *p* < 0.05 considered non-normally distributed) as mean ± SD or median (IQR) unless otherwise stated. Variables with log-normal distributions were log-transformed before analysis. To test for differences between groups and over time, a linear mixed model was used, treating pig ID as a random effect and group, time and group-time interaction as fixed effects. For the ex vivo culture of *P.* aeruginosa, *Pa-*IgY and neutrophils a linear mixed model was used, treating experiment ID as a random effect with *Pa-*IgY dilution and +/- neutrophils as fixed effects. For ex vivo cultures without *Pa-*IgY present, Mann-Whitney U was used. Model assumptions of homoskedasticity and normality of residuals were visually inspected by plotting residuals vs. fitted values and Q-Q plots respectively. Change over time was tested from 0 h and group differences were tested from 1 h. For non-parametric data, where applicable, Kruskal-Wallis was used. Data from flow cytometry was indexed to the values when no *Pa-*IgY was present in the sample and analyzed using Friedman’s ANOVA. A p-value < 0.05 was considered statistically significant, data were analyzed using SPSS (IBM SPSS Statistics for Windows, Version 28.0. IBM Corp) and Statistica (ver 14.0, Dell Inc, Tulsa, USA).

### Results

All pigs that received intravenous *P. aeruginosa* increased their sequential organ failure assessment score by at least 2 points and thus fulfilled the sepsis-3 criteria. The same pigs also had an increase of lactate to above 2 mmol/L, and all but one required an infusion of noradrenaline, all pigs but one therefore fulfilled criteria for septic shock [24]. One pig in the *Pa* + 1 *Pa-*IgY group died after 3 h, one pig in the *Pa* group died after 0.5 h. Both pigs died of circulatory collapse. The data from these pigs were included as planned until their death. There was no significant difference in the bacterial concentration in the dose received by the study groups. However, there was a large variation in the concentration in the administered dose (see Figure S1 in Supplement). Therefore, bacterial concentrations in blood and organ biopsies were analyzed indexed to the concentration in the administered dose.

### Bacterial cultures in the groups receiving *P. aeruginosa*

**Fig. 1:**
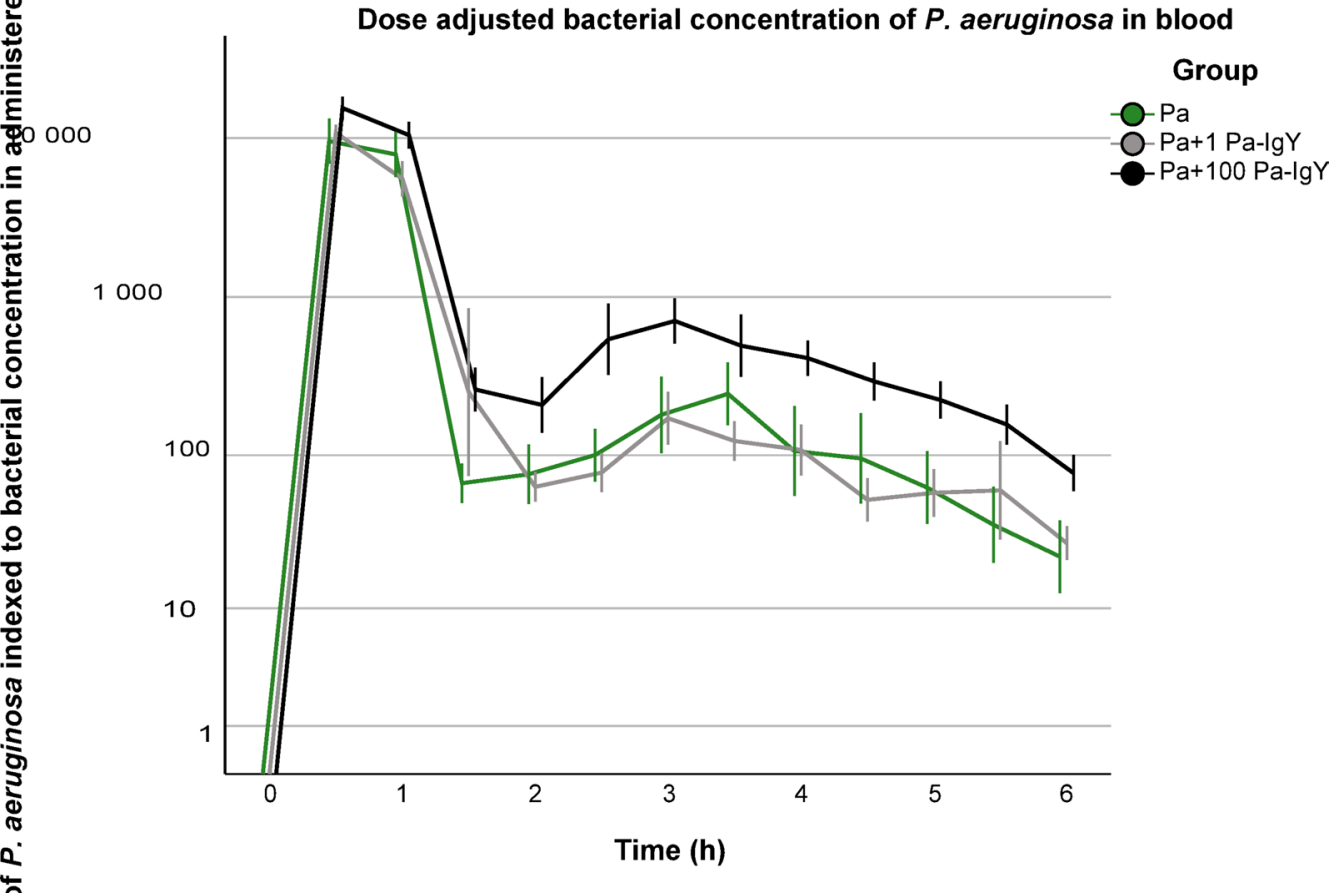
Dose adjusted blood concentration of *P. aeruginosa* over time. Blood concentrations are indexed to the concentration in the administered bacterial dose at the start of the experiment. Values are mean ± standard error. Values were significantly higher over time in the *Pa* + 100 *Pa*-IgY group compared to both other groups (*p* = 0.004). The black line represents *Pa* + 100 *Pa*-IgY, the grey line represents *Pa* + 1 *Pa*-IgY and the green line represents *Pa* group. Values were log-transformed for analysis

Bacterial concentration of *P. aeruginosa* in blood increased over time (*p* < 0.001) in all groups. Values were several times higher in the *Pa* + 100 *Pa-*IgY group compared to the *Pa* and *Pa +* 1 *Pa-*IgY group (*p* = 0.004) (Fig. 1).

**Fig. 2:**
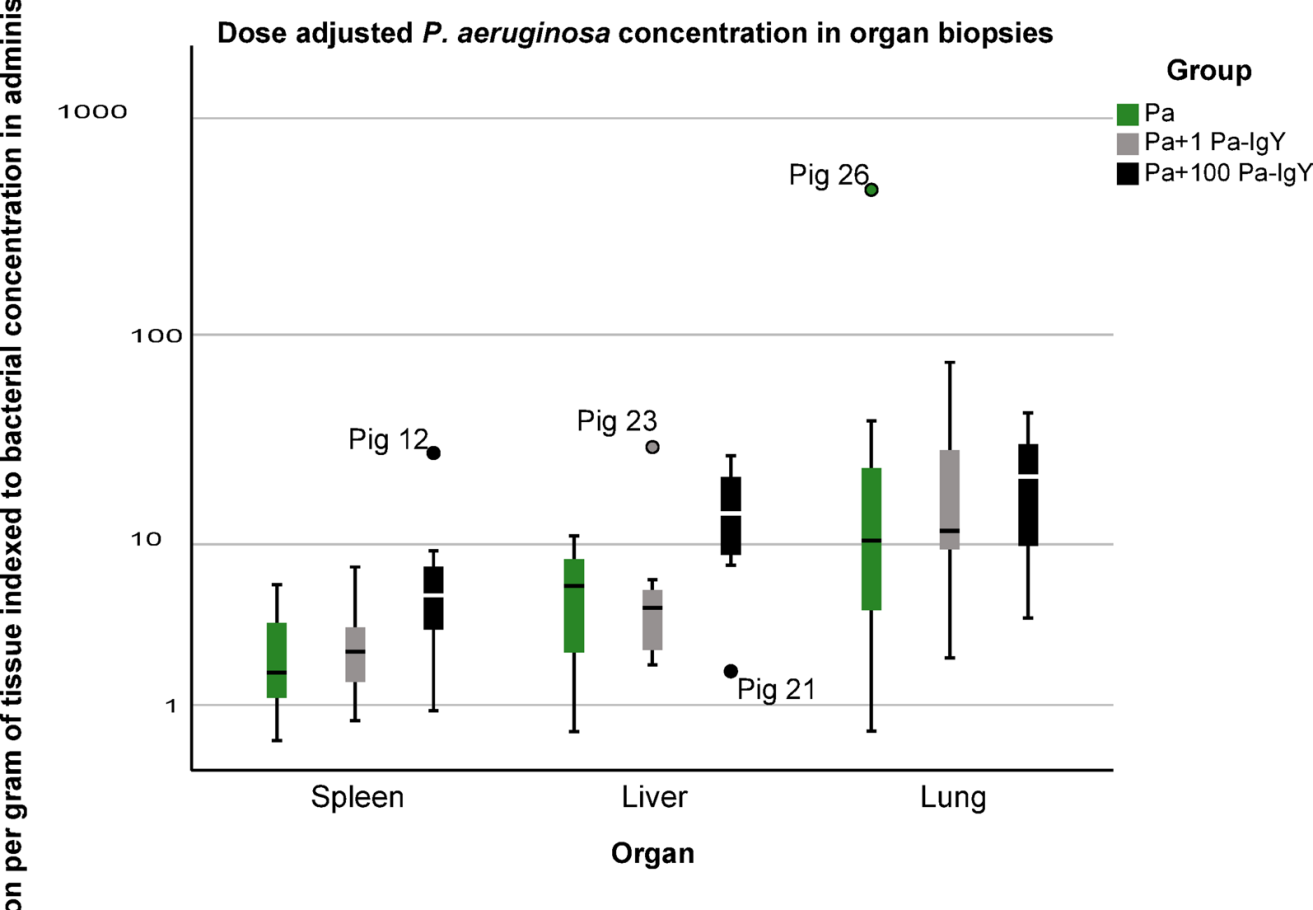
Dose adjusted organ concentration of *P. aeruginosa* in post-mortem biopsies. Organ concentrations are indexed to the concentration in the administered bacterial dose at the start of the experiment. There was no difference between groups. Black represents *Pa* + 100 *Pa*-IgY, grey represents *Pa* + 1 *Pa*-IgY and green represents *Pa* group. Boxplot shows median, IQR, estimated range and the points shows outliers

Post-mortem biopsies of the lungs showed macroscopic signs of illness with widespread atelectasis and pulmonary edema (see Figure S2 in Supplement). Bacterial growth was present in all organ biopsies. There was no difference between groups in bacterial concentration in the lung (*p* = 0.741), the spleen (*p* = 0.100), or the liver (*p* = 0.100) (Fig. 2). Biopsies were performed at 0.5–1 h in one pig from the *Pa* group that died at the beginning of the experiment when blood concentrations of *P. aeruginosa* were high. To test if this has skewed the results a sensitivity analysis was also performed without data from that pig with similar results.

### Culture of Pa-IgY, Neutrophils and *P. aeruginosa*

**Fig. 3:**
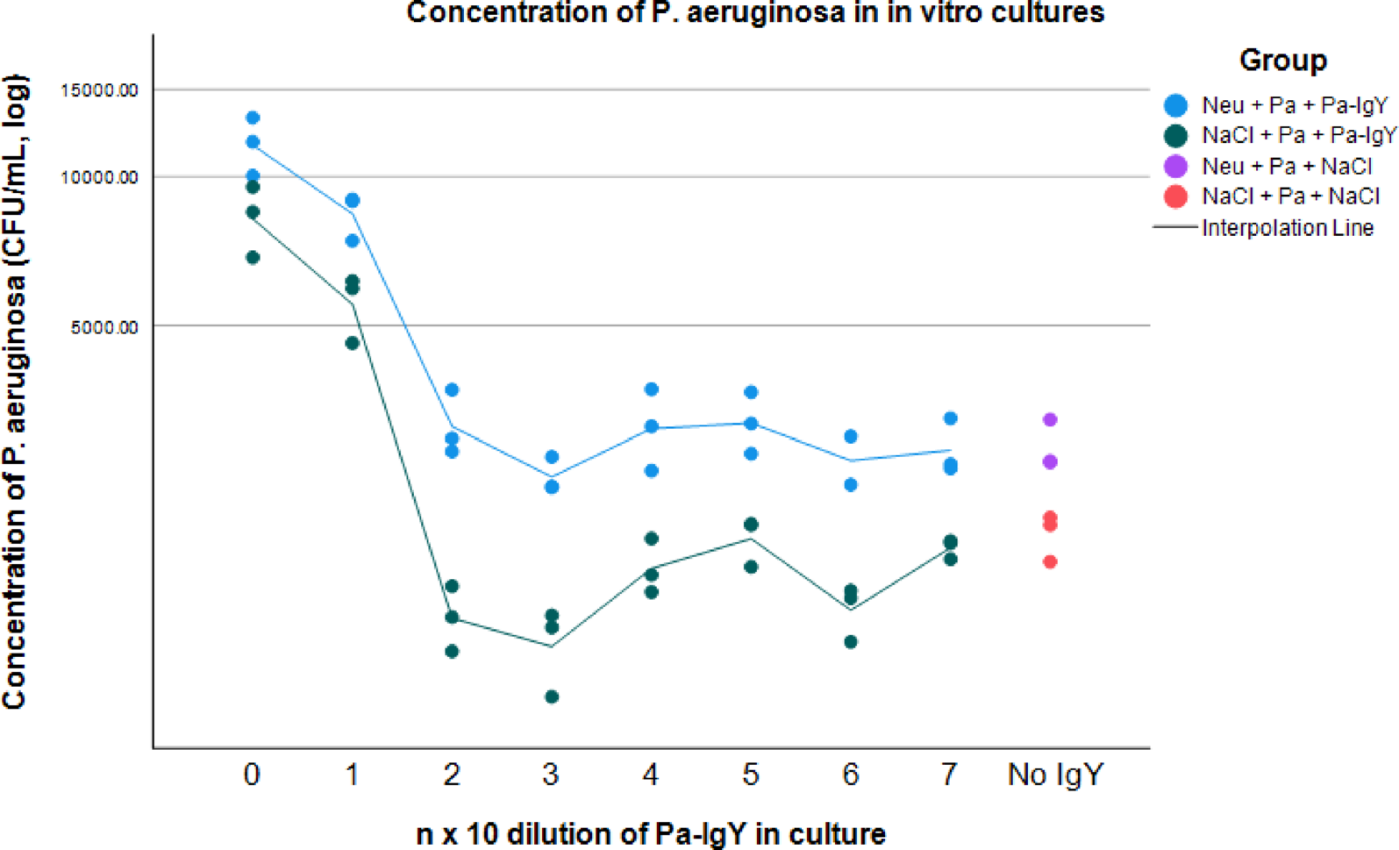
Culture of *P. aeruginosa* +/- *Pa-*IgY +/- neutrophil granulocytes. Values are the number of colonies in bacterial cultures counted using the viable count technique. The x-axis describes how many times *Pa-*IgY was diluted in the culture to create a dose-response curve. *P. aeruginosa* concentration was higher as *Pa-*IgY concentration increased (*p* < 0.001); *P. aeruginosa* concentration was higher in the presence of *Pa-*IgY and neutrophils (blue vs. green line, *p* < 0.001); *P. aeruginosa* concentration was higher in the presence of neutrophils without *Pa*-IgY (purple vs. red dots, *p* = 0.01)

Culture of samples containing *P. aeruginosa* and *Pa-*IgY +/- neutrophils showed a significant effect of *Pa-*IgY dilution on bacterial concentrations (*p* < 0.001). When neutrophils were present in the culture, there was a higher concentration of *P. aeruginosa* compared to when no neutrophils were present (*p* < 0.001). There was also a difference in bacterial concentration in cultures with *P. aeruginosa* +/- neutrophils without *Pa-*IgY present (*p* = 0.01) (Fig. 3).

### Anti *P. aeruginosa* Activity

**Fig. 4:**
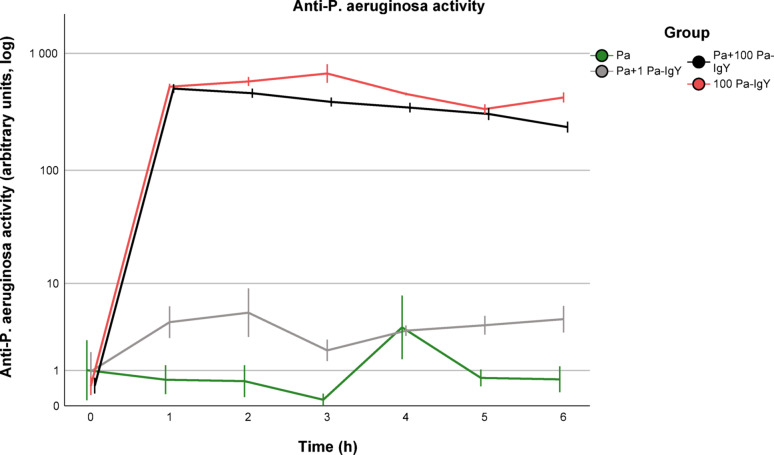
Anti-*P. aeruginosa* activity over time in plasma. Values are mean ± standard error. The black line represents *Pa* + 100 *Pa*-IgY, the grey line represents *Pa* + 1 *Pa*-IgY, the green line represents *Pa* group and the red line represents 100 *Pa-*IgY only. After administration of *Pa-*IgY anti-*P. aeruginosa* activity was highest in the 100 *Pa-*IgY only group (*p* < 0.001) followed by the *Pa +* 100 *Pa-*IgY group (*p* < 0.001) followed by the *Pa +* 1 *Pa-*IgY group (*p* < 0.001), compared to the *Pa* group

Anti-*P. aeruginosa* activity at baseline was below the cut-off in all groups analyzed. After administration of *Pa-*IgY anti-*P. aeruginosa* activity was highest in the *Pa +* 100 *Pa-*IgY group (*p* < 0.001) followed by the *Pa +* 1 *Pa-*IgY group (*p* < 0.001) compared to the *Pa* group. Values in the 100 *Pa-*IgY only group were higher than in the *Pa +* 100 *Pa-*IgY group (*p* < 0.001). Values in the *Pa* group were generally below cut-off (Fig. 4).

### Physiological Parameters and Laboratory Analyses in Animals Receiving *P. aeruginosa*

Heart rate increased over time (*p* < 0.001) with no difference between groups, while MAP decreased over time (*p* < 0.003) with no difference between groups. The doses of medications required for circulatory support were similar between groups (Table 2).

**Table 2:** Doses of medications used for hemodynamic resuscitation. Values are median (IQR). The fluid administered was Ringer’s Lactate. There were no differences between groups

Doses of medications used for circulatory support			
-	Noradrenaline (µg)	Adrenaline (µg)	Total volume of fluid boluses (mL)
Pa	1215 (133–2227)	35 (20–175)	0 (0-770)
Pa + 1 Pa-IgY	1399 (305–2230)	35 (10–40)	495 (0-870)
Pa + 100 Pa-IgY	285 (115–673)	25 (0–40)	0 (0–0)

**Fig. 5:**
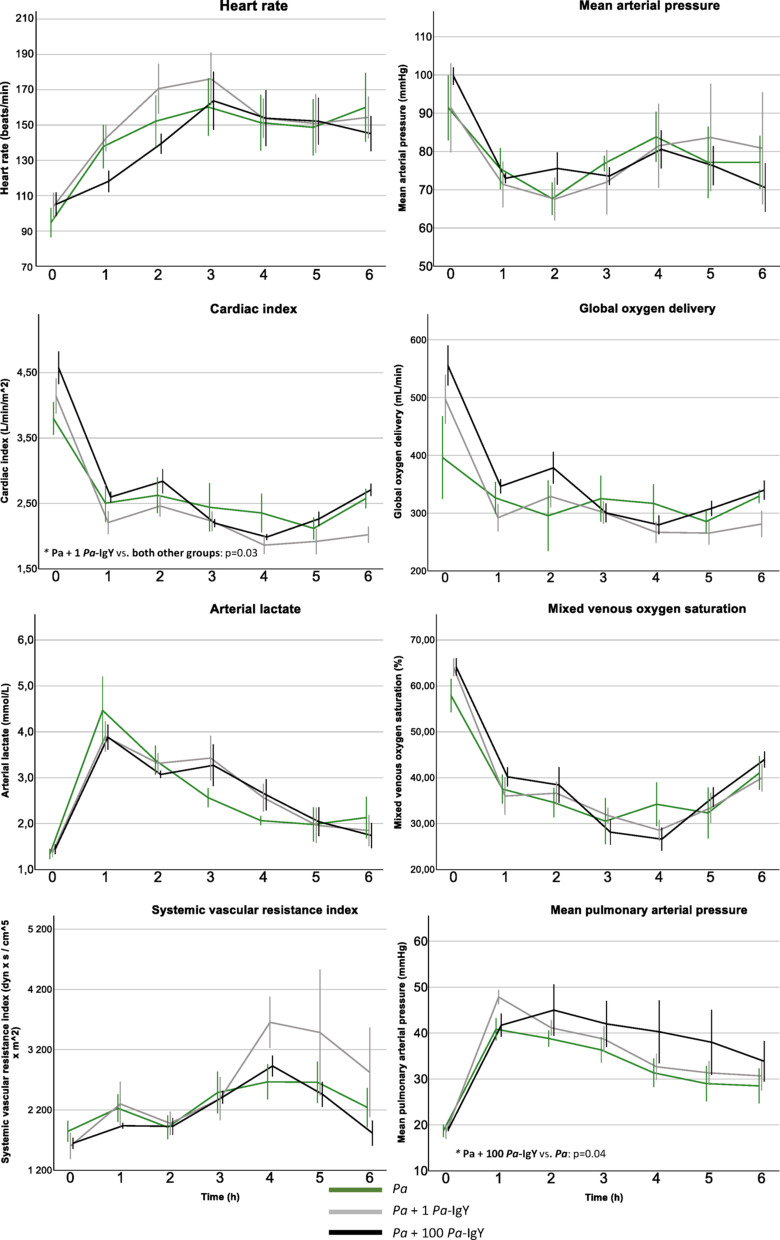

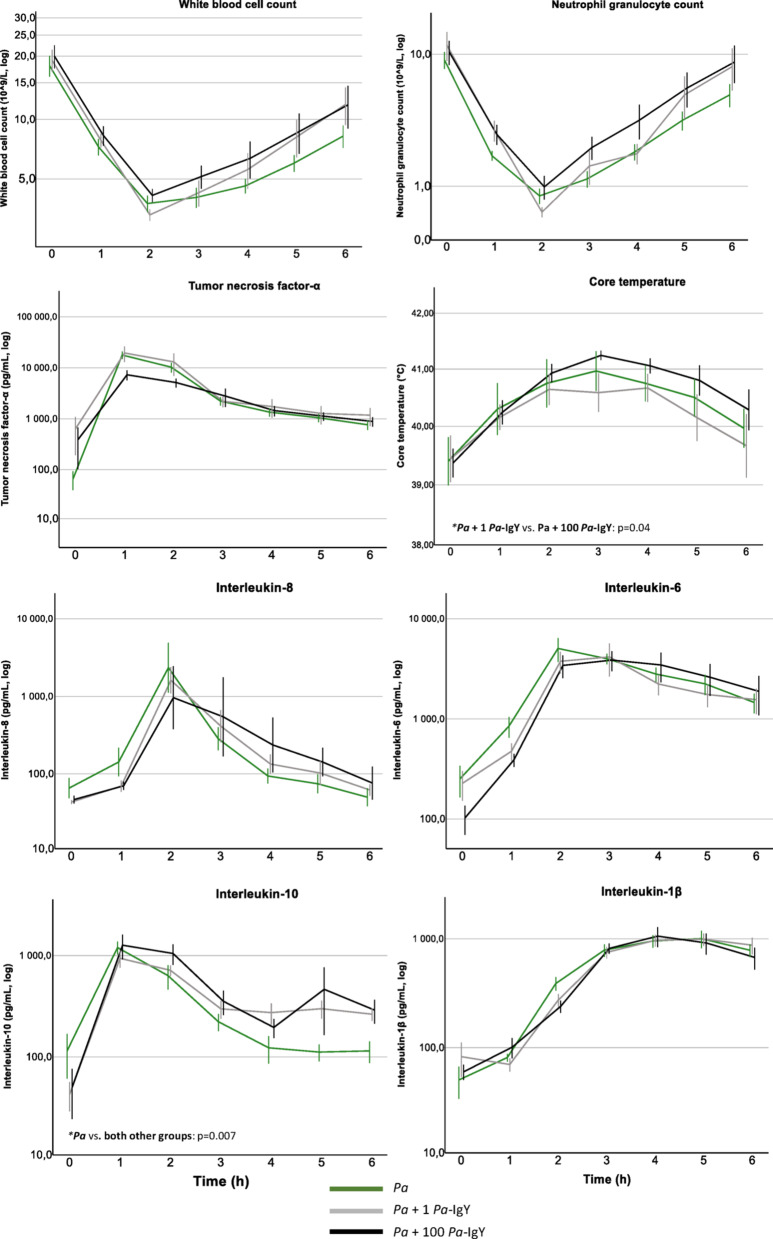

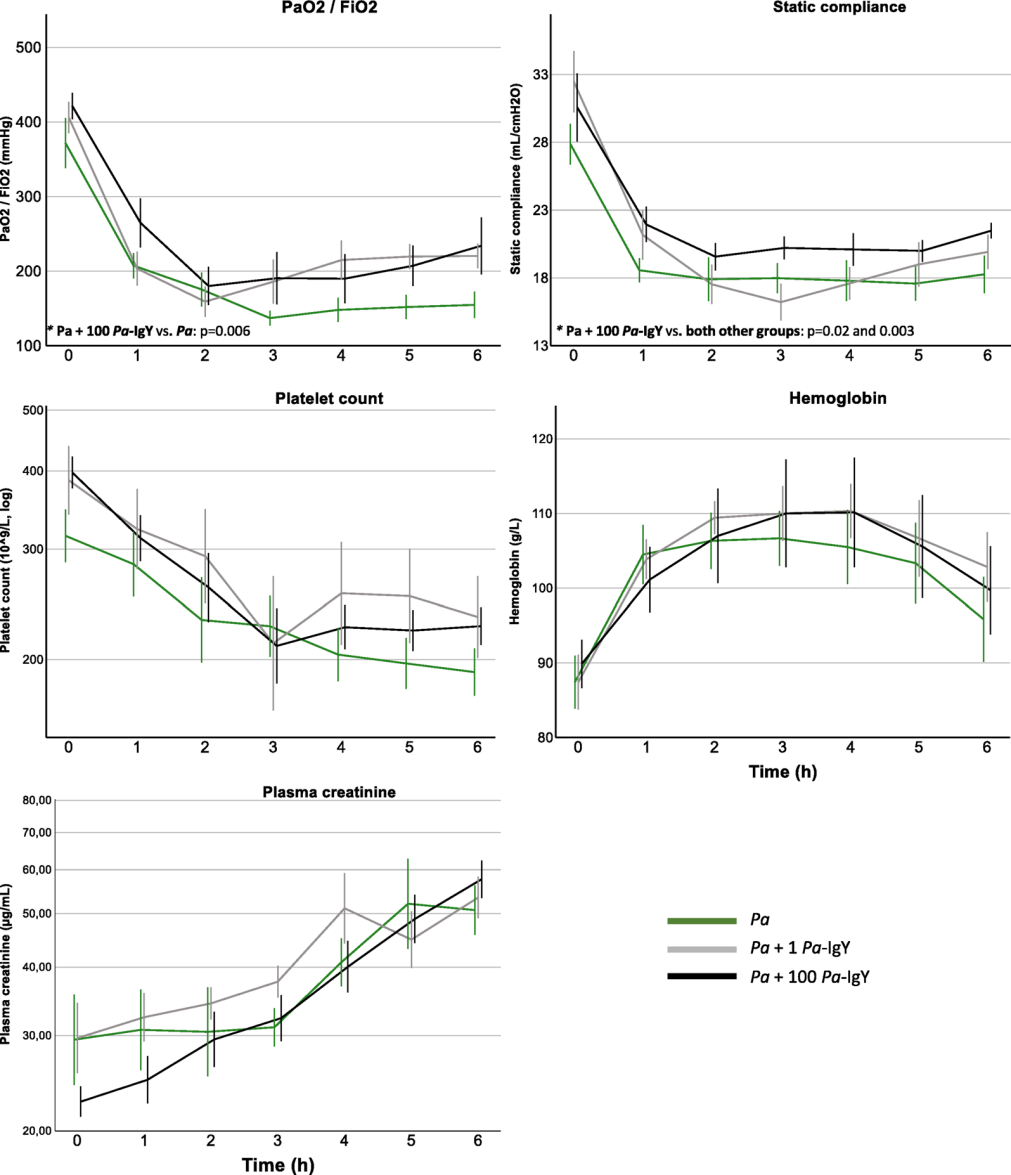
**(a)** Circulatory variables over time. Values are mean ± standard error. *=A significant group difference was found. The black line represents *Pa* + 100 *Pa*-IgY, the grey line represents *Pa* + 1 *Pa*-IgY and the green line represents *Pa* group. All x-axes represent time (h). **(b)** Inflammatory markers over time. Values are mean ± standard error. *=A significant group difference was found. Black line represents *Pa* + 100 *Pa*-IgY, grey line represents *Pa* + 1 *Pa*-IgY and green line represents *Pa* group. All x-axes represent time (h). (**c)** Markers of organ function over time. Values are mean ± standard error. *=A significant group difference was found. The black line represents *Pa* + 100 *Pa*-IgY, the grey line represents *Pa* + 1 *Pa*-IgY and the green line represents *Pa* group. All x-axes represent time (h)

Cardiac index decreased over time (*p* < 0.001) with lower values in the *Pa* + 1 *Pa-*IgY group compared to both other groups (*p* = 0.03). Global delivery of oxygen and mixed venous oxygen saturation (SvO_2_) decreased, while SVRI and lactate increased over time (*p* < 0.009) with no difference between groups. Mean pulmonary arterial pressure (MPAP) increased over time (*p* < 0.001) with higher values in the *Pa +* 100 *Pa-*IgY group compared to the *Pa* group (*p* = 0.04) (Fig. 5a).

Static compliance decreased over time (*p* < 0.001) with higher values in the *Pa* + 100 *Pa-*IgY group compared to the *Pa* + 1 *Pa-*IgY group (*p* = 0.02) and the *Pa* group (*p* = 0.003). PaO_2_/FiO_2_-ratio decreased over time (*p* < 0.001) with higher values in the *Pa* + 100 *Pa-*IgY group compared to the *Pa* group (*p* = 0.006).

Plasma creatinine increased over time (*p* < 0.001) with no difference between groups. Platelet count decreased over time (*p* < 0.001) with no difference between groups. Hemoglobin increased over time (*p* < 0.001) with no difference between groups (Fig. 5b).

Core temperature increased and then decreased over time (*p* < 0.001) with lower values in the *Pa* + 1 *Pa-*IgY group compared to the *Pa* + 100 *Pa-*IgY group (*p* = 0.04). White blood cell count (WBC) and neutrophil granulocyte count (NGC) decreased over time (*p* < 0.001) with no difference between groups. TNF-α, IL-6, IL-8 and IL-1β increased over time (*p* < 0.001) with no difference between groups. IL-10 increased over time (*p* < 0.001) with higher IL-10 levels in the Pa + 1 Pa-IgY group (*p* = 0.007) and Pa + 100 Pa-IgY group (*p* = 0.03) compared to the Pa-only group (*p* = 0.03) (Fig. 5c).

### Results in the high dose Pa-IgY only group

The ’100 *Pa-*IgY only’ group responded to the dose of *Pa-*IgY with an increase in MPAP, resulting in hypotension requiring resuscitation with adrenaline (Figure S3 in supplement). One pig in the *Pa* + 100 *Pa-*IgY group developed asystole after the bolus of *Pa*-IgY but before the start of the bacterial dose (Figure S4 in the Supplement).

### Results in the anaesthesia only group

Results from the anaesthesia only group are found in the Supplemental material.

### Flow cytometry of phagocytosis

There was a large degree of phagocytosis for all dilutions of *Pa-*IgY used with no differences between dilutions.

### Histopathology

The characteristics and grading of histopathological results can be found in the supplemental material (Table S1 + S2).

## Discussion

We have used a porcine model of bacteremia and septic shock caused by *P. aeruginosa* to test the hypothesis that treatment with intravenous *Pa-*IgY reduces the concentration of *P. aeruginosa* in blood. Yet, there was an increase in blood concentration of *P. aeruginosa* when pigs were treated with a high dose of *Pa-*IgY compared to a low dose and to no treatment.

In our model, there was a rapid increase in blood *P. aeruginosa* concentrations with subsequent continuous growth of *P. aeruginosa* in blood cultures throughout the experiment. This likely represents the initial bolus and infusion followed by sustained bacteremia. The initial hours of the experiment were characterized by rapid development of septic shock with subsequent normalization of lactate. Septic shock is a known phenotype of *P. aeruginosa* bloodstream infections in selected patient populations [25]. The pigs also developed respiratory failure corresponding to moderate acute respiratory distress syndrome.

These results contradict the effect previously seen on porcine tracheal colonization by *P. aeruginosa* where *Pa-*IgY decreased the bacterial concentration in large airways [6]. An anti-bacterial effect of IgY was also seen in murine models of *P. aeruginosa* pneumonia when *Pa-*IgY was locally administered [9, 12]. To our knowledge, there are no previous studies of *Pa-*IgY in *P. aeruginosa* bacteremia. *Pa-*IgY is known to efficiently bind antigens and form large immune complexes [26]. In the pathogenesis of *P. aeruginosa* pneumonia, bacteria attach to epithelial surface receptors through adhesins, such as those found in the flagellum and pili of *P. aeruginosa* [27]. Aggregation of bacteria and binding to adhesins by *Pa-*IgY might prevent adhesion and colony formation at the airway epithelium, allowing for clearance by antimicrobial peptides and mucociliary clearance [28]. This is supported by the fact that studies of monoclonal *Pa-*IgY against flagellin and outer membrane protein have shown effect against experimental respiratory infections and - burn wounds, the targets being important for adhesions and biofilm formation respectively [9, 29]. However, in blood, bacterial clearance is dependent on phagocytosis and complement activation, both of which are activated by the Fc-region of immunoglobulins. *Pa-*IgY does not activate the mammalian Fc-receptor and might therefore not promote bacterial clearance in porcine blood [30]. Decreasing ability of *P. aeruginosa* to adhere is unlikely to have an effect on bacterial concentration in bacteremia as *P. aeruginosa* can reproduce in suspension [31]. The 100 *Pa-*IgY only group had higher anti-*P. aeruginosa* activity than the *Pa +* 100 *Pa-*IgY group, indicating that *Pa-*IgY are binding to *P. aeruginosa* and therefore decreasing the detectable fraction of *Pa-*IgY.

The finding that *Pa-*IgY can increase bacterial concentration was also seen when *P. aeruginosa* and *Pa-*IgY were co-cultured in our study, both in the presence and absence of porcine neutrophils. We did not observe an in vitro bactericidal effect of porcine neutrophils. On the contrary, the presence of neutrophils increased the concentration of *P. aeruginosa*. This effect is likely specific to the experimental protocol, as flow cytometry showed phagocytosis of *P. aeruginosa*. Any differences in the degree of phagocytosis between concentrations of *Pa-*IgY are likely not expected, as the protocol allowed for phagocytosis for one hour. During this time, the maximal capacity for phagocytosis is likely reached, even if there are differences in the rate of phagocytosis. Due to the chronology of our experiments and the subsequent discovery of the increase in bacterial concentration, flow cytometry was not performed with the concentrations of *Pa-*IgY that increased bacterial growth. Because of this, did not provide detailed information on the mechanism behind increased bacterial concentrations. It is possible that the physicochemical properties of *Pa-*IgY, other egg yolk proteins present in the *Pa-*IgY solution, or some other factor promotes bacterial growth or decreases bacterial death. The protein content of an IgY-preparation after removal of the lipid phase has been characterized [32]. It contains anti-bacterial proteins (such as ovotransferrin and cystatin) while proteases produced by *P. aeruginosa* might cleave proteins such as ovalbumin to growth-promoting nutrients [33–35]. It is not possible to determine from our experiment how these proteins affect bacterial concentrations. Previous studies have shown that immunoglobulins have an ability to increase bacterial concentrations, especially at high doses [36]. The ex vivo culture bacterial concentrations do not display the typical U-shaped prozone pattern with an intermediate concentration of antibodies displaying an effect. However, it might still be possible that high concentrations of *Pa-*IgY interfere with CFU-enumeration and increase culture concentrations. Another possible explanation is that *Pa-*IgY blocks important epitopes on *P.* aeruginosa impeding clearance by the innate immune system. The exact mechanism behind increased bacterial concentrations is not elucidated by our study and remains unclear.

There was no difference in bacterial concentration in biopsies from the lung, liver and spleen. However, there was a trend towards increased organ bacterial concentrations, most pronounced in splenic biopsies, when high dose *Pa-*IgY was used. This might imply that the same mechanism responsible for increasing bacterial concentrations in blood and in vitro is also present in the organs.

The *Pa* + 100 *Pa-*IgY group had higher MPAP than the *Pa*-group. Porcine sepsis caused by *P. aeruginosa* has been shown to cause pulmonary vasoconstriction [37]. The mechanism of *Pa-*IgY related increase in MPAP is not known. In the 100 *Pa-*IgY only group, we observed an increase in MPAP during the first minutes of the experiment. This effect had dramatic circulatory effects with hypotension and bradycardia in some pigs, requiring resuscitation. The mechanism is not apparent from our experiments. Hyperviscosity syndrome due to immunoglobulins has been reported to cause pulmonary hypertension and it might explain the circulatory effects of *Pa-*IgY [38]. The dose of *Pa-*IgY was given in the central venous catheter as a rapid bolus, which would produce a high initial concentration in the pulmonary vasculature, after which it distributes in the entire distribution volume, and this could explain the transient effect. Although we cannot find studies providing a direct immunological explanation of the phenomenon, avian proteins can be immunogenic in pigs. For example, porcine IgG is produced in response to enteral IgY [39]. The time frame in our study is too short for *Pa-*IgY to cause an adaptive immune response but previous exposure to IgY or egg proteins might sensitize the pigs. Macrophages in the porcine pulmonary vasculature are known to induce pulmonary vasoconstriction in reaction to blood-borne molecules which might also explain the phenomenon [40, 41]. Interestingly, the high dose *Pa* + 100 *Pa-*IgY resulted in better static compliance and PaO_2_/FiO_2_-ratio. This is in accordance with our previous research, where intravenous *Pa-*IgY improved PaO_2_/FiO_2_-ratio in porcine pneumonia, where there was no bacteremia. Our experiment was not designed to investigate mechanisms of pulmonary effect. Increased pulmonary function could imply that *Pa-*IgY can modify the inflammatory pulmonary injury caused in pneumonia as well as in septic shock and/or bacteremia. However, this is highly speculative and contradicted by the fact that inflammatory markers were similar between groups. Perhaps the pulmonary vasoconstriction from *Pa*-IgY augments the effects of hypoxic pulmonary vasoconstriction, resulting in better PaO_2_/FiO_2_-ratio. The effect on static compliance could also be due to the observed pulmonary vascular effects with decreased permeability and therefore decreased pulmonary exudate. A low dose of *Pa-*IgY resulted in higher CI. Since the effect on CI was seen in the low-dose *Pa-*IgY group and no other signs of a parabolic effect of *Pa-*IgY on circulatory function are evident, this finding might be the result of type 1 error.

Interestingly, the increase in bacterial concentration in the *Pa +* 100 *Pa-*IgY group did not result in a greater inflammatory response. There was no difference in inflammatory cytokines implies that *Pa-*IgY had no major effects on reducing inflammation. However, IL-10 was lower when pigs were treated with a high dose of *Pa-*IgY, which indicates that *Pa-*IgY might affect the inflammatory response in pigs. As it is an isolated finding, it might be the result of type 1 error.

A strength of this model is that it reliably produces septic shock and respiratory failure using live bacteria. We wanted to study *Pa-*IgY in a model that resembles the patients seen in the ICU, as these patients are at risk of *P. aeruginosa* infections with a high mortality. A risk of using this model is that the illness is too severe for *Pa-*IgY to have an effect on our secondary endpoints. Therefore, small effects of *Pa-*IgY on our secondary endpoints might be difficult to study in this model. The number of pigs in this study was low, increasing the fragility of the results. Limiting the number of animals is important from an ethical perspective. The study did not use a fully blinded design, which may have increased the risk of observer bias. To limit this risk, the commercially affiliated authors did not have influence over the planning or conduction of animal experiments. The analysis of anti-*P. aeruginosa* activity was performed blinded to group allocation. In addition, primary and secondary endpoints were assessed using objective measurements rather than subjective assessments. There was large variability in the *P. aeruginosa* concentration of the administered bacterial dose, albeit without significant differences between groups. Measuring optical density of a sample to estimate bacterial concentration is standard practice and allows for fast, inexpensive and simple standardization of cell concentrations [42]. However, it is an indirect measure of cell concentration and can be affected by, for example, varying aggregation of *P. aeruginosa* in solution [43]. Although the procedure of culturing *P. aeruginosa* was standardized, it is conceivable that it results in varying degrees of both virulence and viability [44].

The results from this study introduce complexity to the use of non-mammalian antibodies as antimicrobial agents. The potential of *Pa-*IgY to increase bacterial concentrations shold be considered in future research. The mechanism of increased bacterial concentrations both in vivo and in vitro was not elucidated by our data. Future strategies to decrease virulence and promote bacterial killing in *P. aeruginosa* bacteremia could consist of monoclonal antibodies directed at antigens of specific importance to the virulence of *P. aeruginosa* in blood or fusion of mammalian Fc-complexes to *Pa*-IgY for opsonization and complement activation. To further investigate the safety of *Pa*-IgY, the mechanism behind the increase in MPAP needs to be investigated along with a safe dosing regimen. This is the second porcine study to show that there are potential lung protective effects of *Pa*-IgY in models of P. aeruginosa infections. Future research should examine whether this is mediated through an effect on the pathogen or through an effect on the lungs themselves.

## Conclusions

In this study of porcine septic shock caused by *P. aeruginosa* bacteremia, *Pa-*IgY increased the concentrations of *P. aeruginosa* in blood. The results were supported by in vitro findings.

## Electronic Supplementary Material

Supplementary material 1

## Data Availability

The datasets used and/or analysed during the current study are available from the corresponding author on reasonable request.
