# Supplementary material for: Intravenous anti-P. aeruginosa IgY antibodies increase bacterial concentration in porcine septic shock with bacteremia
Source: Intensive Care Med Exp. 2026 Aug 19;14:108. doi: 10.1186/s40635-026-00952-y (PMC13486454; doi:10.1186/s40635-026-00952-y)
Supplement: Supplementary file 1 [file 40635_2026_952_MOESM1_ESM.docx]

Supplementary materials:

Figure S1. Scanning electron microscopy of pre-opsonized P. aeruginosa with Pa-IgY and neutrophils

Figure S2. Concentration of P. aeruginosa in the bacterial dose¬ administered to pigs.

Figure S3. The right lung removed post mortem of a pig that received P. aeruginosa.

Figure S4. Mean pressure in the pulmonary and systemic circulation of pigs in 100 Pa-IgY group.

Figure S5. Heart rate and mean pulmonary arterial pressure over time of one pig in the Pa + 100 Pa-IgY group after receiving Pa-IgY but before the bacterial dose.

Table S1. Flow cytometry of FITC-marked P. aeruginosa ex vivo Flow cytometry of FITC-marked P. aeruginosa ex vivo

Table S2. Histopathological results

Table S3. Variables of the group receiving anesthesia only.

Supplement

Method of flow cytometry

For opsonization to occur, 100 µL of FITC-labeled *P. aeruginosa* 10^5^ CFU/mL was incubated with 100 µL NaCl 9 mg/mL (control sample) or with 100 µL *Pa*-IgY 22.5 mg/mL diluted 10^3^, 10^4^, 10^5^, 10^6^ or 10^7^ (based on a preliminary results from the co-culture of neutrophils, *Pa-*IgY and *P.* aeruginosa) times for one hour at 37°C. To each sample, 500 µL of heparinized porcine blood was added, and the samples were incubated for another hour for phagocytosis to occur. After incubation, 100 µL of the samples were immediately added to test tubes in an ice bath containing 100 µL of trypan blue 0.4 % and 1 mL of cold phosphate-buffered saline. The tubes were centrifuged at 4°C for 5 minutes at 400 x g. The supernatant was removed, 3 mL lysing solution (0.15 mol/L NH_4_Cl, 10 mM/L KHCO3, 0.1 mM/L EDTA) was added, and the tubes were left on ice for 15-20 minutes. The tubes were then centrifuged at 4°C for 5 minutes at 400 x g and the supernatant was removed. The remaining leukocytes were washed once with 1 mL PBS and thereafter suspended in 500 µL PBS. Granulocyte phagocytosis was measured by detection of intracellular FITC-labelled Pseudomonas, where the cellular fluorescence is related to the number of bacteria per cell. Flow cytometry was performed by a Navios flow cytometer (Beckman Coulter, Miami, FL, USA). Granulocytes were identified by their forward-side scatter dot plot profile and the mean fluorescence intensity of granulocytes was measured. The experiment was repeated three times with blood from three different pigs. Due to batch-to-batch variations in the analysis the data was indexed to FITC-expression in the control sample.

Results of flow cytometry

When assessing neutrophil phagocytosis using FITC-labelled *P. aeruginosa,* there was no difference in the mean FITC-signal in neutrophils across the different concentrations of *Pa-*IgY (p=0.8) (Table 3). A FITC-signal was found in 93-99 % of all neutrophils in the sample. Neutrophil count in the different samples was 7.8, 6.9 and 6.3 x 10^9^/L, respectively.

**Table S1.** Flow cytometry of FITC-marked *P. aeruginosa ex vivo*. Values are mean ± SD. There were no differences between groups.

| Group | Mean FITC-signal indexed to the *P. aeruginosa* sample |
| --- | --- |
| *P. aeruginosa* | 1.00 ± 0.00 |
| *P. aeruginosa* + *Pa-*IgY diluted 10^7^ times | 0.97 ± 0.87 |
| *P. aeruginosa* + *Pa-*IgY diluted 10^6^ times | 1.02 ± 0.12 |
| *P. aeruginosa* + *Pa-*IgY diluted 10^5^ times | 0.98 ± 0.09 |
| *P. aeruginosa* + *Pa-*IgY diluted 10^4^ times | 1.02 ± 0.16 |
| *P. aeruginosa* + *Pa-*IgY diluted 10^3^ times | 1.02 ± 0.10 |

Method of electron microscopy

The solution was fixated by centrifuging at 4000g for 20 minutes and then dissolving the pellet in 2.5% glutaraldehyde and 150mM sodium cacodylate. The samples were washed with cacodylate buffer and dehydrated with an ascending ethanol series from 50% (vol/vol) to absolute ethanol. The specimens were then subjected to critical-point drying with carbon dioxide, and absolute ethanol was used as an intermediate solvent. The tissue samples were mounted on aluminum holders, sputtered with 20nm palladium/gold, and examined in a DELPHI (PhenomWorld Delmic, Netherlands) correlative light and scanning electron microscope.

Results of electron microscopy

Scanning electron microscopy showed both activated and inactive neutrophils. There were sparse instances of neutrophil extracellular traps, and phagocytosis occurred, but was rare. *P. aeruginosa* was often seen in large aggregates (Figure S1).

**Figure S1.** Scanning electron microscopy of pre-opsonized *P. aeruginosa* with *Pa-*IgY and neutrophils. The left panel shows an aggregate of *P. aeruginosa*. The right panel shows an activated neutrophil granulocyte and a single *P. aeruginosa*.

**Method for histopathological analysis**

Biopsies were immediately fixed in 4% formaldehyde. The samples were then trimmed, processed to paraffin, cut at 4µ using a standard microtome (Thermo Scientific mod. Microm HM355S, UK) and were routinely stained with haematoxylin and eosin. For examination and photography, a Nikon microscope (mod. Eclipse E600, Japan) equipped with a digital microscope color camera (The Imagine Source mod. DFK NME33UX264, Germany) was used.

**Table S2.** Semi-quantitative grading of histopathological findings.

**Figure S2.** Concentration of *P. aeruginosa* in the bacterial dose­ administered to pigs. *P. aeruginosa* was given intravenously as a 10 mL bolus dose followed by an infusion of 0.3 mL/kg/h for 1 hour. There was no significant difference between groups.

**Figure S3.** The right lung removed post mortem of a pig that received *P. aeruginosa*. The macroscopic changes seen were fluid-filled alveoli, hemorrhage and atelectasis. The image and severity are representative of the pigs receiving *P. aeruginosa*.

**Figure S4.** Mean pressure in the pulmonary and systemic circulation of pigs in 100 *Pa-*IgY group Values are mean and error bars represent standard error. The blue line is the mean pulmonary arterial pressure (left axis) and the red line is the mean arterial pressure (right axis).

**Figure S5.** Heart rate and mean pulmonary arterial pressure over time of one pig in the *Pa* + 100 *Pa-*IgY group after receiving *Pa*-IgY but before the bacterial dose. *Pa-*IgY was administered at time = 0. The pig developed asystole one minute after administration of *Pa-*IgY.

**Table S3.** Variables of the group receiving anesthesia only. Values are mean ± SD or median (IQR) according to normality.

| **Time:** | **0** | **1** | **2** | **3** | **4** | **5** | **6** |
| --- | --- | --- | --- | --- | --- | --- | --- |
| Heart rate (beats/min) | 94±14 | 80±13 | 84±16 | 83±15 | 81(77-99) | 88±12 | 83±12 |
| Mean arterial pressure (mmHg) | 87±8 | 82(77-84) | 82(73-85) | 78±12 | 80±11 | 74(70-90) | 83(71-94) |
| Lactate (mmol/L) | 1.8±0.6 | 1.3±0.7 | 1.6±0.3 | 1.3±0.5 | 1.3±0.8 | 1.2±0.8 | 1.0±0.2 |
| Core temperature (°C) | 38.6±0.7 | 39.0±0.9 | 39.1±0.9 | 39.3±0.8 | 39.8(38.9-40.3) | 39.8±0.8 | 39.9±0.8 |
| White blood cell count (10^9^/L) | 19.9 (15.0-24.3 | 21.3±5.4 | 22.9±4.4 | 23.5±5.3 | 23.8±5.2 | 21.1±3.9 | 21.2±3.3 |
| PaO_2_/FiO_2_ (mmHg) | 420±72 | 440±18 | 429±34 | 394±60 | 420±46 | 419±55 | 358±48 |
| Static compliance (mL/cmH_2_O) | 32.3±7.8 | 29.3±4.8 | 25.6(24.0-32.1) | 26.1±6.1 | 23.2±5.2 | 23.2±3.5 | 23.7±4.6 |
